# Supplementary material for: Predictors for the Occurrence of Seizures in Meningioma
Source: Cancers (Basel). 2024 Aug 31;16(17):3046. doi: 10.3390/cancers16173046 (PMC11394441; doi:10.3390/cancers16173046)
Supplement: Supplementary file 1 [file cancers-16-03046-s001.zip › cancers-3124102-supplementary.pdf]

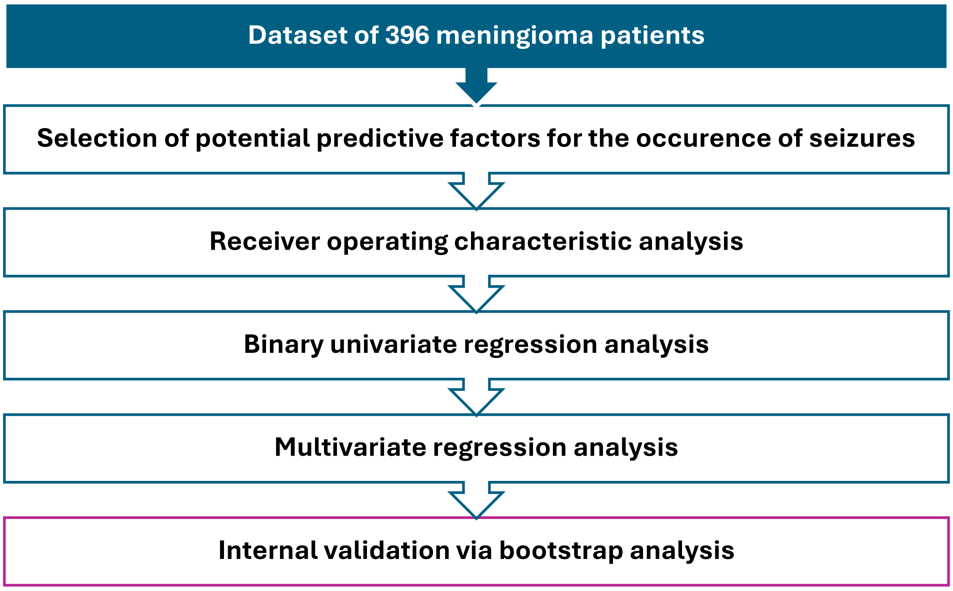

Figure S1. Flow chart of the statistical analysis process for identifying independent predictive factors for the occurrence of seizures in meningioma patients.

A

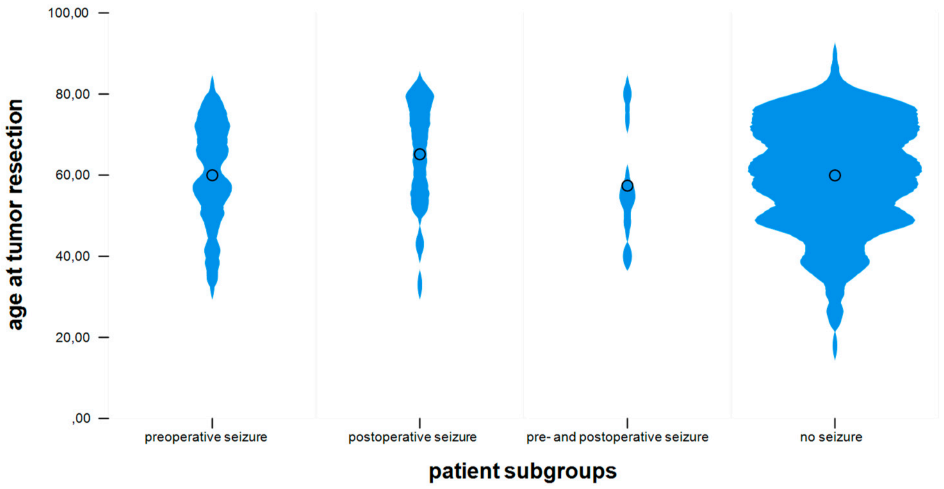

patient subgroups	N	Mean	Std. Deviation	Minimum	Maximum
preoperative seizure	52	59.98	12.54	33.00	81.00
postoperative seizure	31	65.16	12.86	33.00	82.00
pre- and postoperative seizure	12	57.42	13.83	40.00	81.00
no seizure	301	59.94	13.37	18.00	89.00
Total	396	60.28	13.27	18.00	89.00

B

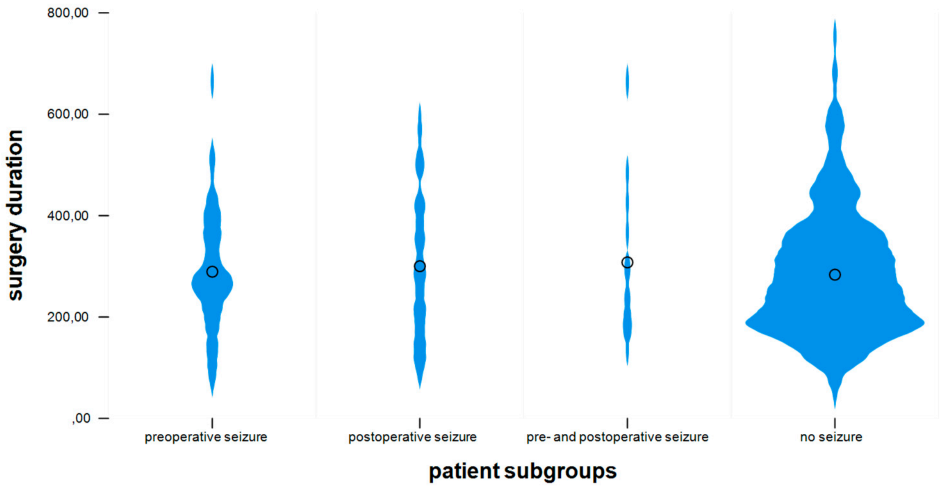

patient subgroups	N	Mean	Std. Deviation	Minimum	Maximum
preoperative seizure	52	289.38	115.24	77.00	665.00
postoperative seizure	31	300.29	141.92	92.00	588.00
pre- and postoperative seizure	12	307.83	154.00	138.00	664.00
no seizure	301	283.50	121.51	53.00	753.00
Total	396	286.32	123.11	53.00	753.00

C

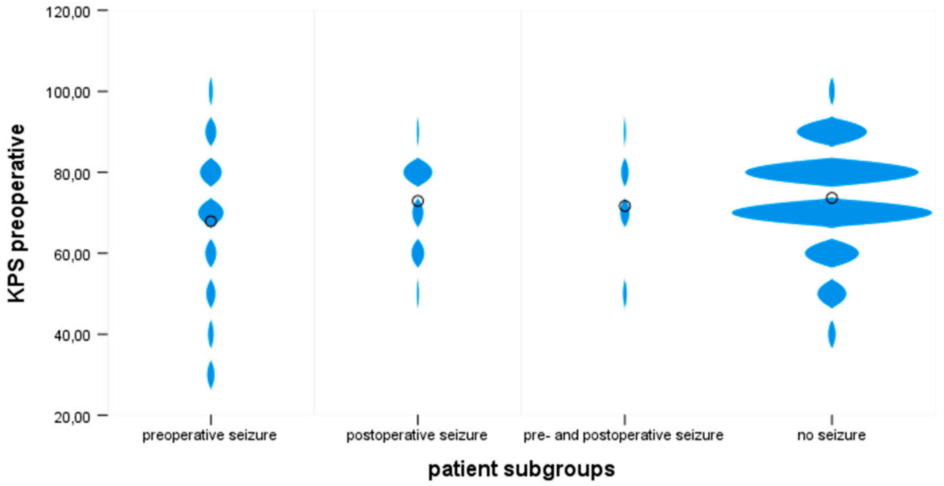

patient subgroups	N	Mean	Std. Deviation	Minimum	Maximum
preoperative seizure	52	67.88	18.19	30.00	100.00
postoperative seizure	31	72.90	9.73	50.00	90.00
pre- and postoperative seizure	12	71.67	11.93	50.00	90.00
no seizure	301	73.65	11.10	40.00	100.00
Total	396	72.78	12.30	30.00	100.00

Figure S2. Demographical visualizations of patients' characteristics (A) age at tumor resection, (B) surgery duration and (C) preoperative KPS.
Abbreviations: KPS, Karnofsky Performance Score; Std., Standard.

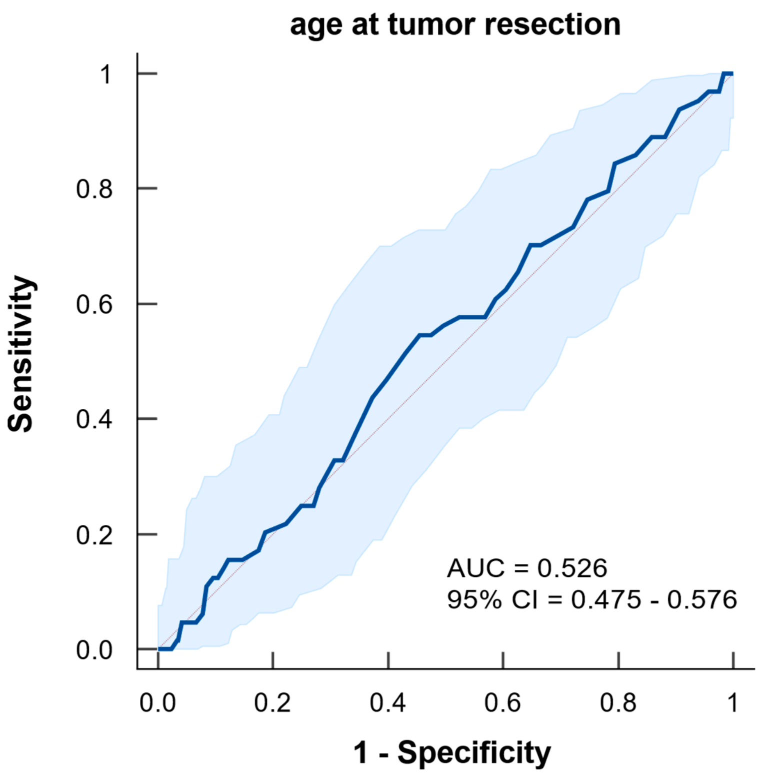

Figure S3. ROC analysis with 95% confidence interval of the parameter age at tumor resection for the occurrence of preoperative seizures in meningioma patients.
Abbreviations: ROC, receiver operating characteristic; AUC, area under the curve; CI, confidence interval.

A

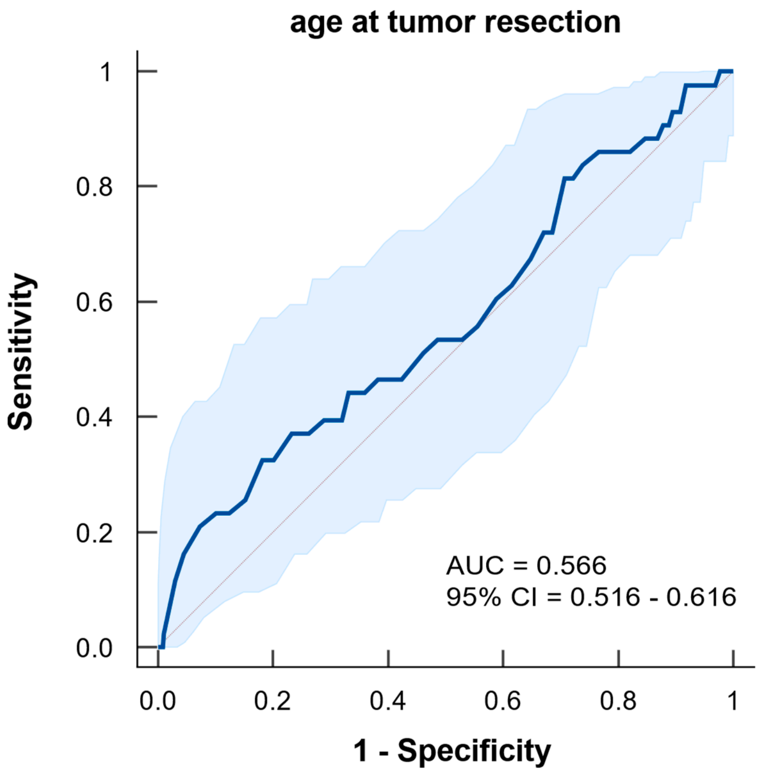

B

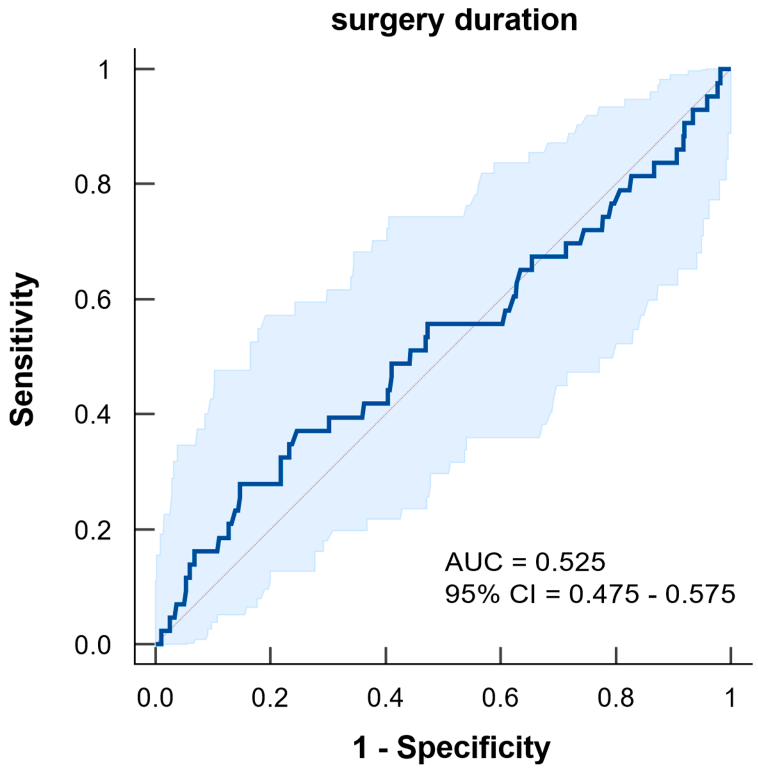

C

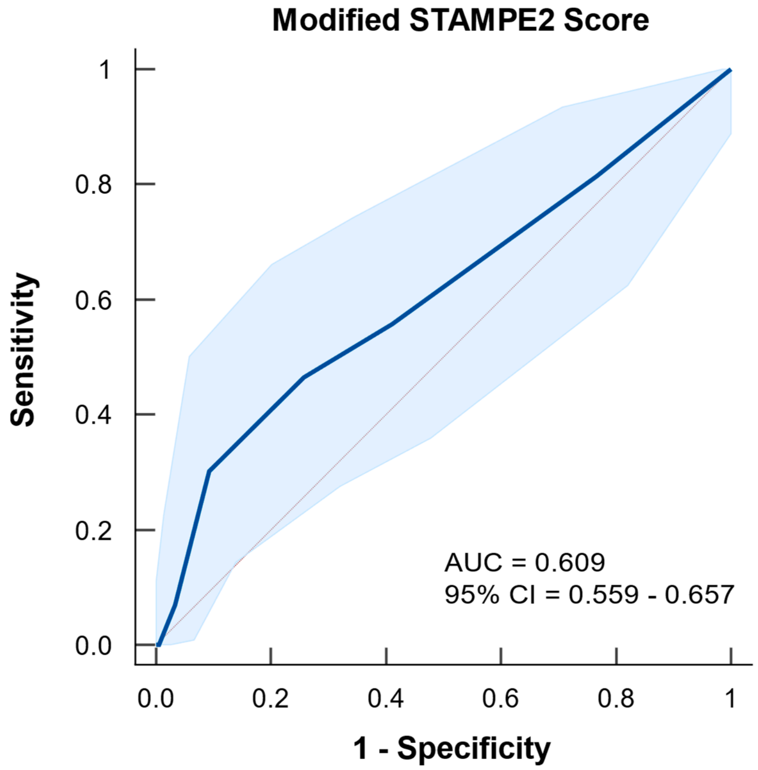

Figure S4. ROC analyses with 95% confidence intervals of the parameters (A) age at tumor resection, (B) surgery duration and (C) modified STAMPE2 score for the occurrence of postoperative seizures in meningioma patients.

Abbreviations: ROC, receiver operating characteristic; AUC, area under the curve; CI, confidence interval.

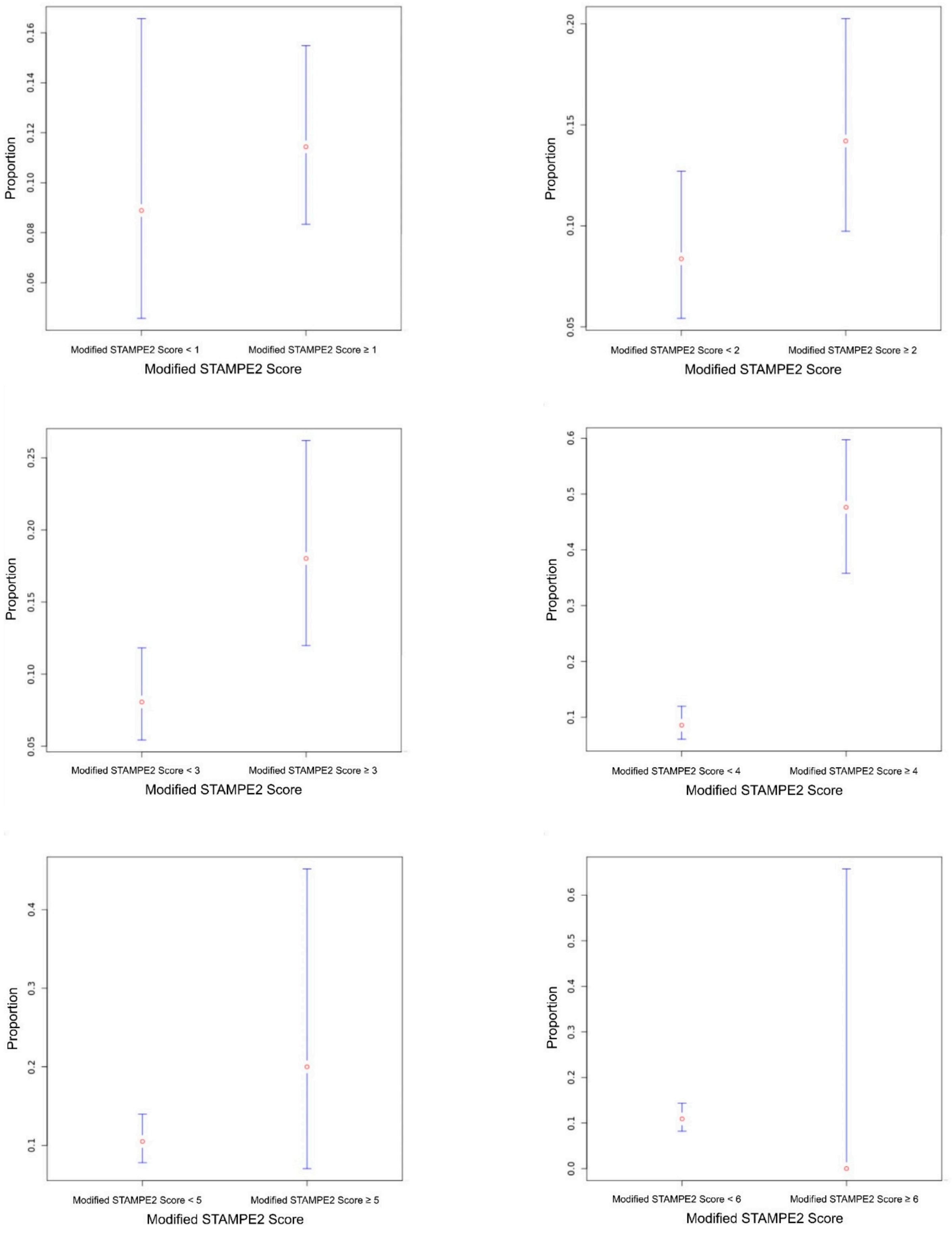

Figure S5. Proportions with 95% confidence intervals of patients with postoperative seizures in the study population dichotomized into different cut-off values of the modified STAMPE2 score.

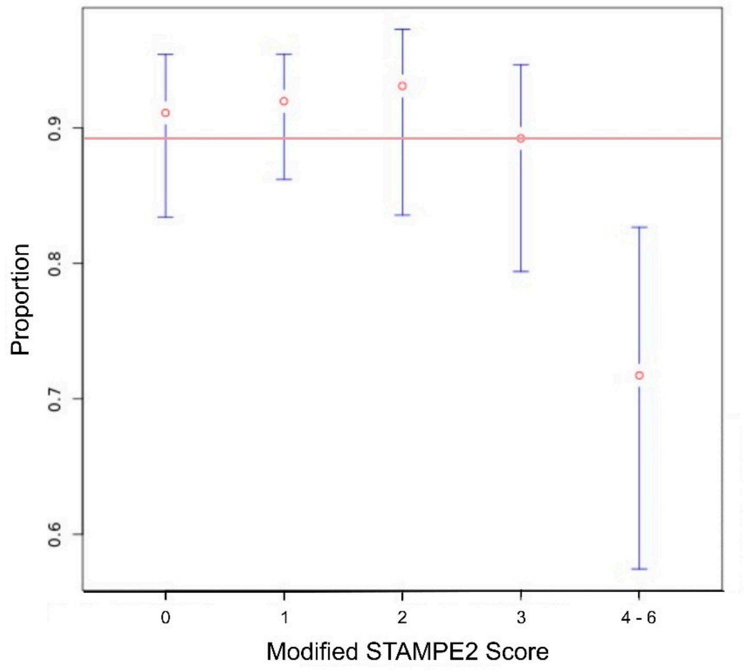

Figure S6. Proportions with 95% confidence intervals of patients with no postoperative seizures stratified by different values of the modified STAMPE2 score. The horizontal red line shows the proportion of patients with no postoperative seizures in the entire study population.
